# Flipped classroom improves nursing students’ theoretical learning in China: A meta-analysis

**DOI:** 10.1371/journal.pone.0237926

**Published:** 2020-08-27

**Authors:** Bao-Zhu Li, Nv-Wei Cao, Chun-Xia Ren, Xiu-Jie Chu, Hao-Yue Zhou, Biao Guo

**Affiliations:** 1 Department of Epidemiology and Biostatistics, School of Public Health, Anhui Medical University, Hefei, Anhui, China; 2 Anhui Province Key Laboratory of Major Autoimmune Diseases, Hefei, Anhui, China; 3 Department of Surgery, The First Affiliated Hospital of Anhui Medical University, Hefei, Anhui, P.R. China; 4 Department of Human Resource, The Second Affiliated Hospital of Anhui Medical University, Anhui, Hefei, China; University of Edinburgh, UNITED KINGDOM

## Abstract

**Objective:**

At present, current didactic teaching delivery method help nursing students apply theory to clinical situations in an inefficient way. The flipped classroom (FC), a novel teaching mode emphasizing self-study and critical thinking, has generated interest in nursing education in China. However, there are a gap in the literature and no consistent outcomes of current studies which compared FC and lecture-based learning (LBL), and no systematic review has comprehensively compared theoretical scores as an affected outcome in FC versus LBL modes.

**Methods:**

In this review, we analyze flipped-learning nursing students’ scores, and aim to assess the efficacy and provide a deeper understanding of the FC in nursing education. Following the inclusion criteria, articles were obtained by searching PubMed, Embase and Chinese data, including the China National Knowledge Infrastructure, Wanfang Data, and VIP database until 3 January 2020. Data were extracted from eligible articles and quality was assessed. A meta-analysis was then performed using a random effects model with a standardized mean value (SMD) and a 95% confidence interval (CI).32 studies were included after reviewing 2,439 citations. All studies were randomized controlled trials (RCTs). The FC theoretical knowledge scores in FC were significantly positively affected compared to those of the traditional classroom (SMD = 1.33, 95% CI: 1.02–1.64; *P* < 0.001). In addition, 23 studies reported skill scores, indicating significant difference between the FC mode and LBL mode (SMD = 1.58, 95%CI: 1.23–1.93; *P* < 0.001).

**Conclusions:**

The results of this meta-analysis suggest that compared to the LBL teaching method, the FC mode dose significantly improve Chinese nursing students’ theoretical scores. However, the problems of heterogeneity and publication bias in this study need to be remedied high-quality future studies.

## Introduction

At present, nursing is taught mainly via the traditional teaching mode, which is teacher-centered. In traditional lectures, teachers often use a stand-and-deliver approach, in which students passively receive lesson-related information [1]. In China, teachers generally adopt this traditional teaching mode, which is a common didactic method, with course material delivered by book. Also called lecture-based learning (LBL), this traditional teaching method has obvious shortcomings. As cognitive subjects, students passively receive knowledge during the teaching process, leading to their insufficient grasp of professional knowledge and a lack of enthusiasm for learning. Although the traditional mode helps students master practical skills in a short time, it cannot promote thinking and discussion among students toward the overarching goal of cultivating their abilities [2]. Consequently, exploring a new teaching mode to promote students’ acquisition of knowledge and skills has become an important current topic.

In the early 1990s, Eric Mazur proposed the peer teaching method, the earliest form of flipping the classroom [3]. In 1996, Maureen J. Lage and Glenn J. Platt proposed the idea of inverting the classroom [4]. In 2000, Baker recommended using online tools to guide classroom teaching [5]. In 2004, Salman Khan uploaded short instructional videos online. Bergmann and Sams [6], two high school chemistry teachers, started FCs in 2007. Moreover, founded in 2007, the Khan Academy provided online teaching services for the first time. They prerecorded video lectures as homework assignments for their students so that class time could be dedicated to problem-solving activities and the review of difficult materials [6]. The FC is a novel teaching method that differs from traditional teaching. The focus on the FC is the student's accountability for their own learning, and a necessary reflective approach. The FC also entails a process that shifts learning from memory recall to comprehension and application [6–8]. Its advent indicates that education is undergoing a role shift, from teacher- (teaching) to student-oriented (characterized by active student participation) teaching strategies [9]. This novel teaching method alters the amount of time that is spent inside versus outside the traditional classroom, placing students in control of their time. Before class, students engage in independent learning by reading the handout(s) or watching the video materials that the teacher has provided. Students then inform the teacher if they have problems with the material. In class, the teacher and the students discuss any problematic aspects together [10].

Nurses constitute one of the important components of the healthcare system. At present, the world is experiencing a serious epidemic caused by the coronavirus disease 2019 (COVID-19), and there is an extreme shortage of medical workers. A large volume of medical workers went to assist with the COVID-19 impact, but their numbers were still insufficient. Nurses are a necessary element of the treatment process. To perform their roles, nurses were required substantial professional knowledge to face a variety of potential problems. As such, nursing teaching is of great significance in contemporary medical education. For nurses, the skill of critical thinking is essential to provide safe and comprehensive care [11]. However, the current didactic teaching delivery method does not help nursing students foster the ability to reason critically and apply theory to clinical situations [12]. Therefore, scholars have begun to seeking other effective methods to promote student enthusiasm and efficiency in nursing teaching [2, 13–15]. Since the FC can enhance students’ understanding of materials through active learning, it has recently become a popular, effective teaching model for application to health professions [16], medical [17], dental [18], and pharmaceutical education [19]. However, the existing studies have not strictly treated the randomized controlled trial (RCT) as an inclusion criterion [20–22]. RCT is well-equipped to deal with the intervention factors’ effects on outcomes, thereby reducing selection bias and other potential bias. Excluding non-RCTs is a great way to improve the quality of the meta-analysis.

The FC has been widely applied in various nursing education courses. There has been a systematic review, with a meta-analysis indicating that the flipped approach positively affects nursing students’ skill competence [22]. Since theory is the basis of practice, faculties usually set up theory courses in advance of practical operation to teach theoretical knowledge and furnish a theoretical basis. Students need to translate theoretical knowledge into practice to improve their nursing abilities. At present, there is a lack of research to comprehensively analyze flipped-learning nursing students’ theoretical test scores in RCTs. It is therefore necessary to conduct a meta-analysis to quantitatively analyze existing evidence in RCTs. In this review, we analyze flipped-learning nursing students’ scores, and aim to assess the efficacy and provide a deeper understanding of the FC in nursing education.

## Methods

### Search strategy

A systematic search was carried out in PubMed, Embase and Chinese database including the China National Knowledge Infrastructure, Wanfang Data, and VIP database. The search was from database establishment to the end of 3 January 2020. The search strategy was used as follows: (flipped OR inverted) AND (class OR learn) AND (nursing OR nurse teaching). Examples of search strategies are as follows: 1) The search strings were run in PubMed: (((flipped OR inverted)) AND (class OR learn)) AND (nursing[MeSH Terms] OR nurse education); 2) For Embase we used the strings: (flipped* OR inverted*) AND (class* OR learn*) AND (nursing* OR 'nurse education'); 3) In Chinese databases, the search terms were used as (flipped classroom OR inverted classroom) AND nursing.

### Inclusion criteria

Abstracts of all articles were used for preliminary screening, and obtained full articles for further screening. The inclusion criteria that met requirements were as follows: 1) The study must be designed as a randomized controlled trial; 2) The subjects were nursing undergraduates, higher and middle vocational students in schools rather than in hospitals; 3) The course of the study was related to nursing. 4) The experimental group received flipped teaching, consisting self-learning by video before class and problem-solving activities in class; 5) The control group received traditional lecture-based learning (LBL) method which was typically used in general classroom; 6) Objective-based assessment, such as theoretical knowledge test, was applied to evaluate students’ learning outcomes and quantitative theory score was taken as an outcome; 7) Published articles were in Chinese. Articles would be excluded as long as met one of the criteria: 1) The study wasn’t reported as a randomized controlled trial; 2) The subjects were interns or nurses in hospitals; 3) The control group did not use traditional teaching; 4) It didn’t evaluate the theoretical scores as primary outcomes or had incomplete data; 5) The full text could not be obtained; 6) It was duplicate.

Two searchers (CXR and XJC) screened the articles respectively. All results after screening would be taken into account. All discrepancies were discussed and determined by a third party.

### Data extraction and quality assessment

Relevant information in the included articles was extracted by two researchers independently. If the data is inconsistent, two researchers decided whether to include it after discussing and reaching a consensus. The information was extracted in a table which mainly included first author, publication year, educational background of subjects, disciplines or curricula, duration of intervention, sample size of the experimental group and control group, and test score index. The potential risk of bias was assessed by multiple researchers using the Cochrane Collaboration’s tool [23] independently.

### Statistical analysis

In this study, Review Manager 5.3 (The Cochrane Collaboration, Copenhagen, Denmark) and STATA 14.0 software (STATA Corporation, College Station, TX, USA) were used to work with data. The standardized mean value (SMD) with 95% confidence interval (CI) was used as the effect measure to compare consecutive results from different scales to calculate each study. The point estimates associated with the summary results for each study were shown in forest plots. Heterogeneity of meta-analysis was evaluated by *I*^*2*^ statistic. And *I*^*2*^ statistic with values >50% indicated significant heterogeneity in this study. SMD was combined with 95% CI by using random effect model with heterogeneity or fixed effect model without heterogeneity. If heterogeneity existed, a sensitivity analysis was performed to investigate the impact of each study on the overall estimate. Publication bias was detected by the funnel plots using STATA 14.0 software.

## Results

### Search results

**Fig 1:**
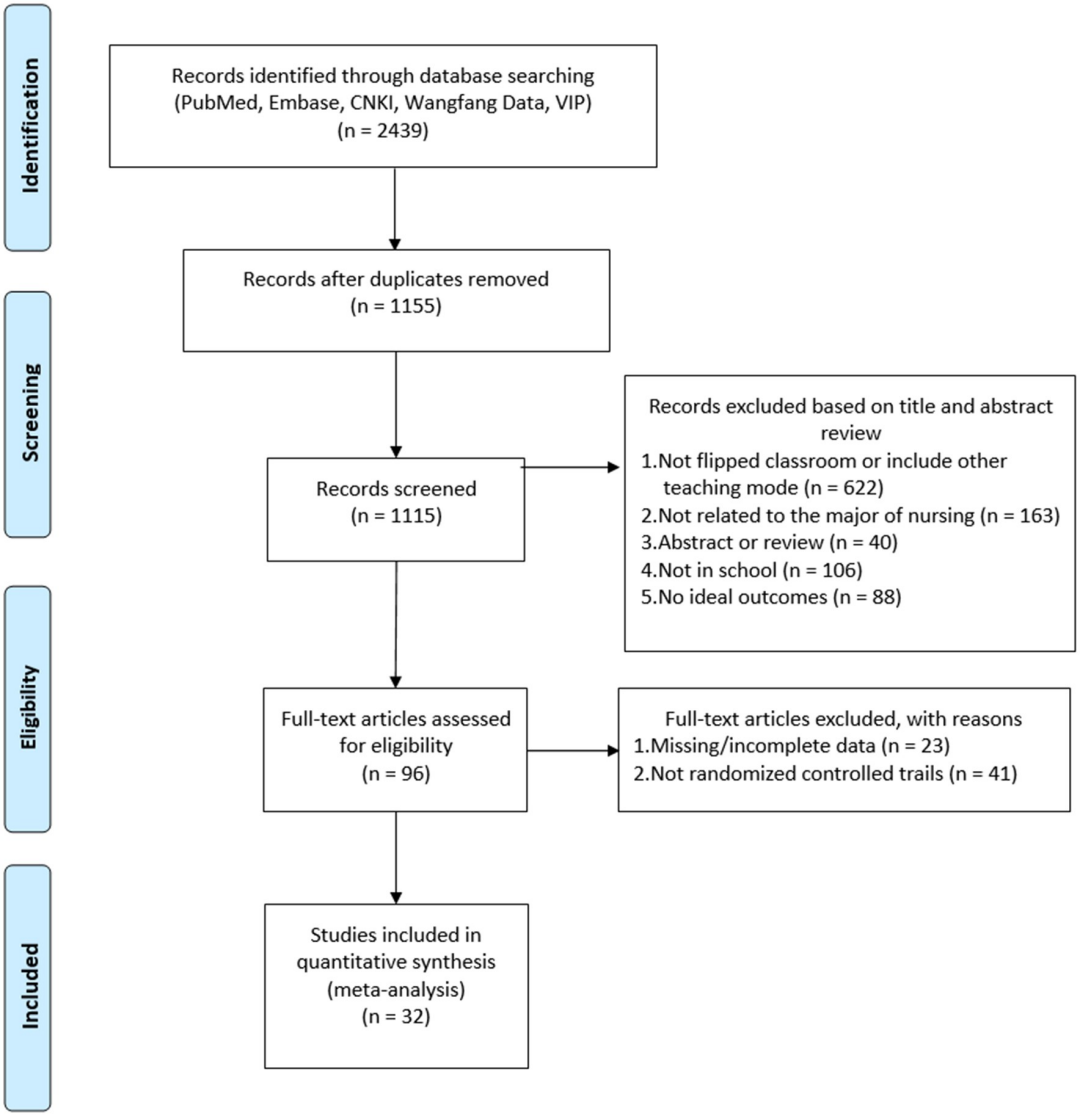
PRISMA diagram for the literature search. CNKI, China National Knowledge Infrastructure.

After systematically searching related databases, a total of 2,439 records were retrieved. After 1,284 records were removed because of duplication, 1,155 records remained. A total of 1,059 records were excluded after screening titles and abstracts. Moreover, 96 records were further screened according to the established inclusion criteria. After reading the full text, 41 records were excluded as non-randomized controlled trials and 23 because of missing or incomplete results. Consequently, only 32 records were included in this meta-analysis. The flow of articles, screened according to the exclusion and inclusion criteria is shown in Fig 1.

### Study characteristics and study quality

**Fig 2:**
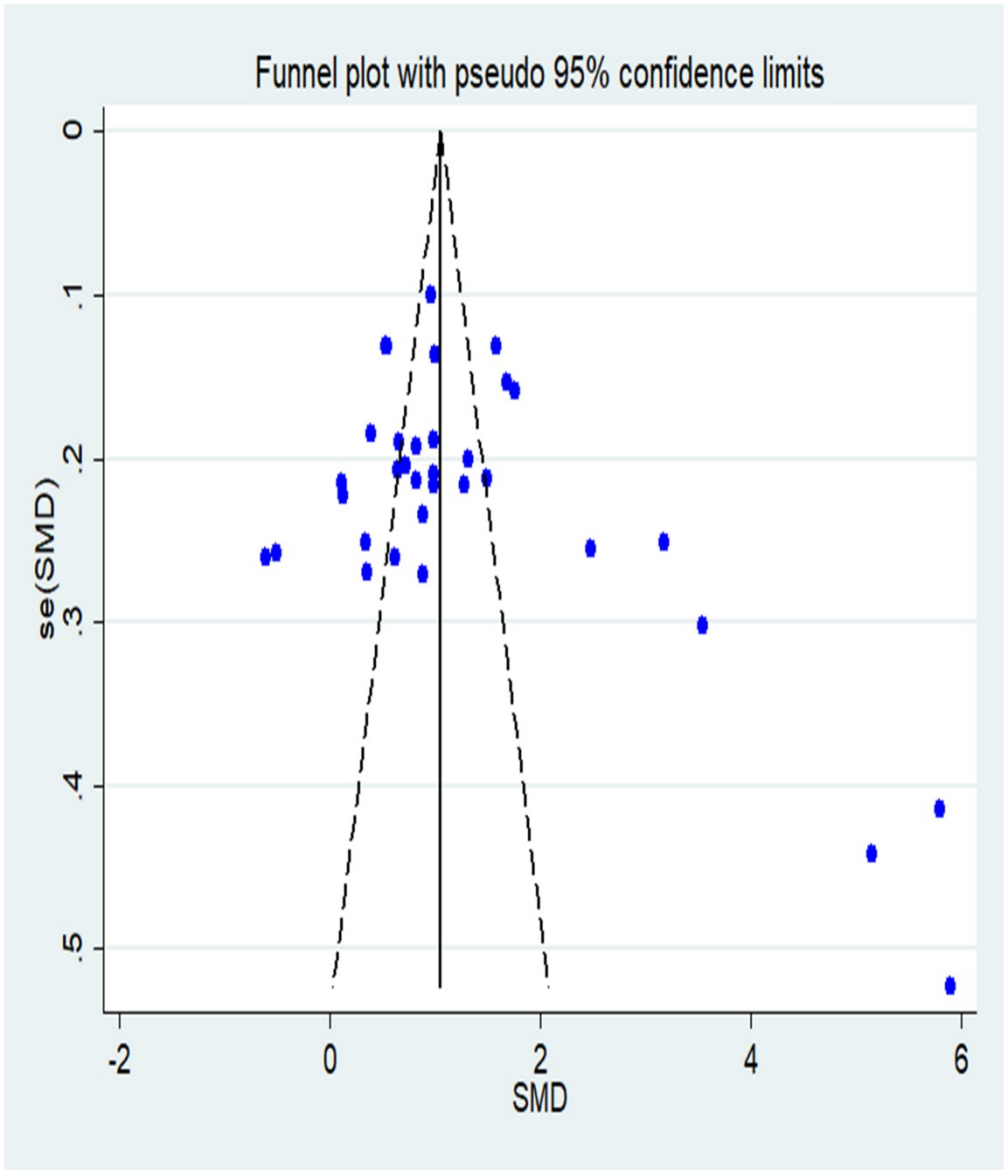
Funnel plot analysis of theoretical knowledge scores for the potential publication bias in the meta-analysis.

**Fig 3:**
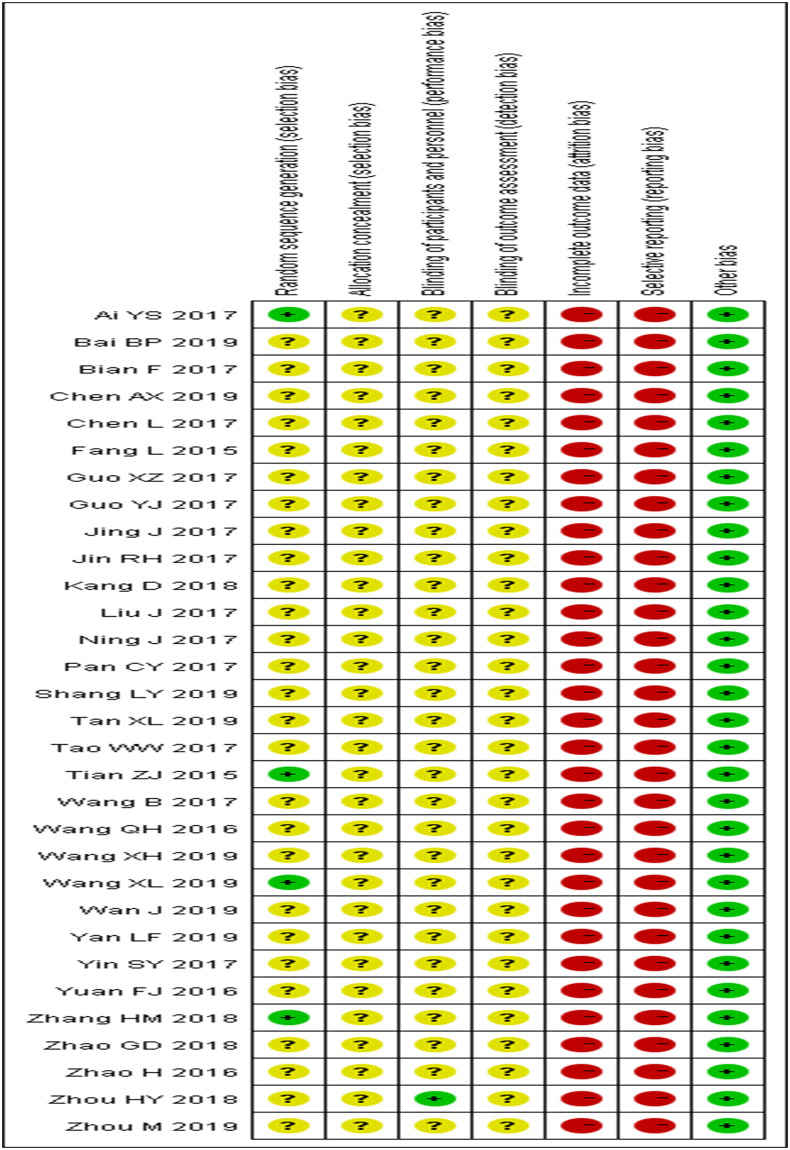
Risk of bias summary.

In total, 32 articles [24–55] were included. These were published between 2015 and 2019, with theoretical scores as comparable outcomes. One included three curricula on the same research object. These studies’ authors are all Chinese, and all articles have been published in Chinese journals. The studies involved 4,389 participants, of which 2,197 and 2,192 belonged to the FC and LBL groups, respectively. The sample size varied from 56 to 448 participants. The subjects in 23 studies [24–44] were undergraduates, while those in 11 studies [45–55] were higher and secondary vocational students. Nine studies [31, 33, 34, 38, 41, 42, 45, 50, 52] applied the FC mode to basic nursing teaching. Four [26, 31, 36, 53] among the included articles compared the two teaching methods in surgical nursing. Three studies [30, 37, 54] used the FC in obstetrics and gynecology nursing. Another three [31, 47, 55] concentrated on internal medicine nursing. The length of the teaching intervention varied from 24 class hours to two semesters. The knowledge examination scores were used to evaluate students’ theoretical knowledge. The baseline characteristics of the included studies are shown in Table 1. Funnel plots of the theoretical knowledge scores were used to assess publication bias, which was found to be present (Fig 2) The risk of bias in all included articles is shown in Fig 3. All included studies were RCTs, but only four illustrated their randomized sequence generation methods. None of the studies describe their allocation concealment and the blinding of outcome assessments. Only one reported applying the blinding process to participants and personnel. None of these studies were conducted in a way to indicate whether data were missing or excluded and whether there had been selective reporting.

**Table 1:** The detailed baseline characteristics of all included studies.

Study ID	Publication year	Study design	Educational background	Disciplines or curricula	Duration of intervention	No. of FC	No. of LBL	Outcomes measures
Wan J [24]	2019	RCT	undergraduate	pathology and pathophysiology	NA	29	27	TS, SS
Shang LY [25]	2019	RCT	undergraduate	critical care nursing	NA	30	30	TS
Wang XL [26]	2019	RCT	undergraduate	surgical nursing	NA	46	50	TS, SS
Bai BP [27]	2019	RCT	undergraduate	practical teaching of health assessment	55 class hours	54	55	TS, SS
Wang XH [28]	2019	RCT	undergraduate	clinical anatomy	NA	63	60	TS, SS
Tan XL [29]	2019	RCT	undergraduate	community nursing	24 class hours	55	59	TS
Chen AX [30]	2019	RCT	undergraduate	obstetrics and gynecology nursing	54 class hours	113	130	TS
Tao WW [31]	2017	RCT	undergraduate	internal medicine nursing	4 months	32	30	TS
Tao WW [31]	2017	RCT	undergraduate	surgical nursing	5 months	32	30	TS
Tao WW [31]	2017	RCT	undergraduate	basic nursing	6 months	32	30	TS
Yin SY [32]	2017	RCT	undergraduate	preventive medicine	54 class hours	40	40	TS
Zhang HM [33]	2017	RCT	undergraduate	basic nursing	28 class hours	49	46	TS, SS
Jin RH [34]	2017	RCT	undergraduate	basic nursing	28 class hours	62	58	TS, SS
Bian F [35]	2017	RCT	undergraduate	nursing research	one semester	72	72	TS, SS
Liu J [36]	2017	RCT	undergraduate	surgical nursing	two semesters	110	110	TS, SS
Guo XZ [37]	2017	RCT	undergraduate	obstetrics and gynecology nursing	one semester	120	120	TS, SS
Ning J [38]	2017	RCT	undergraduate	basic nursing	two semesters	118	122	TS, SS
Zhang Y [39]	2017	RCT	undergraduate	medical statistics	two semesters	158	149	TS, SS
Fang L [40]	2016	RCT	undergraduate	introduction to nursing	NA	53	51	TS
Study ID	Publication year	Study design	Educational background	Disciplines or curricula	Duration of intervention	No. of FC	No. of LBL	Outcomes measures
Yuan FJ [41]	2016	RCT	undergraduate	basic nursing	NA	45	45	TS, SS
Zhao H [42]	2016	RCT	undergraduate	basic nursing	two semesters	115	114	TS, SS
Wang QH [43]	2016	RCT	undergraduate	nursing research	24 class hours	230	218	TS
Tian ZJ [44]	2015	RCT	undergraduate	geriatric nursing	one semester	32	32	TS
Yan LF [45]	2019	RCT	higher vocational education	basic nursing	10 months	58	59	TS, SS
Ai YS [46]	2017	RCT	higher vocational education	emergency nursing	NA	48	50	TS, SS
Chen L [47]	2017	RCT	higher vocational education	internal medicine nursing	NA	51	51	TS, SS
Jing J [48]	2017	RCT	higher vocational education	health assessment technology	NA	50	52	TS, SS
Zhou M [49]	2019	RCT	higher vocational education	pediatric nursing	60 class hours	58	59	TS, SS
Zhou HY [50]	2018	RCT	higher vocational education	basic nursing	NA	40	40	TS, SS
Zhao GD [51]	2018	RCT	higher vocational education	geriatric nursing	38 class hours	40	41	TS, SS
Kang D [52]	2018	RCT	higher vocational education	basic nursing	150 class hours	43	44	TS, SS
Wang B [53]	2017	RCT	higher vocational education	surgical nursing	NA	64	57	TS, SS
Guo YJ [54]	2017	RCT	higher vocational education	obstetrics and gynecology nursing	NA	56	58	TS, SS
Pan CY [55]	2017	RCT	higher vocational education	internal medicine nursing	9 weeks	63	63	TS

FC = flipped classroom, LBL = lecture-based learning, TS = theoretical score, SS = skill scores.

### The effects of FC on theoretical knowledge scores

**Fig 4:**
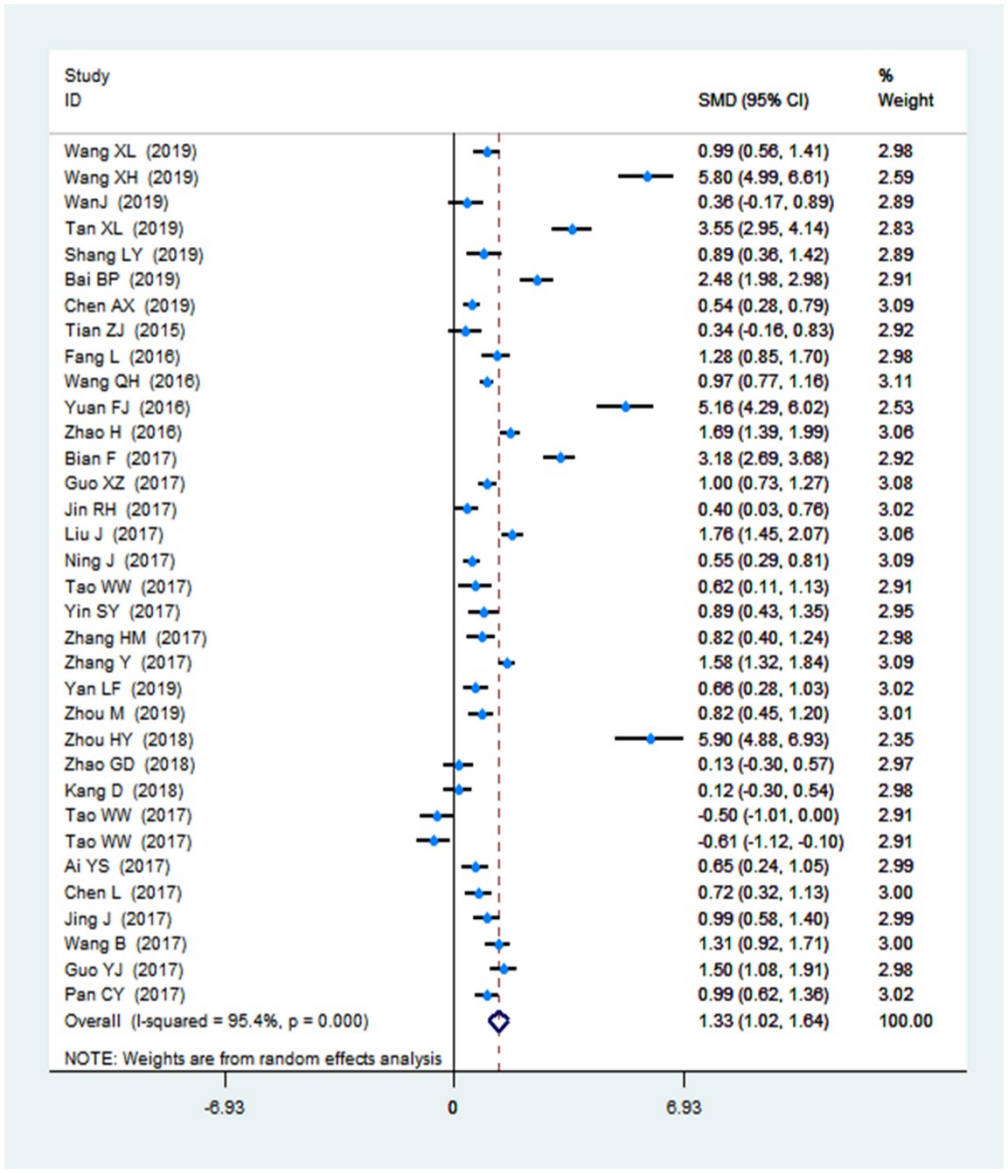
Forest plot of the nursing students' theoretical scores.

Theoretical knowledge scores were reported in all the included studies (2197 and 2192 in the FC and LBL groups, respectively). The mean of the theoretical knowledge scores in the FC was 1.33 compared to the traditional classroom (95%CI: 1.02–1.64; *P* < 0.001). A meta-analysis of a random model was carried out in this study. A forest plot of the nursing students' theoretical score is shown in Fig 4.

### The effects of the FC on the subgroup analyses

**Fig 5:**
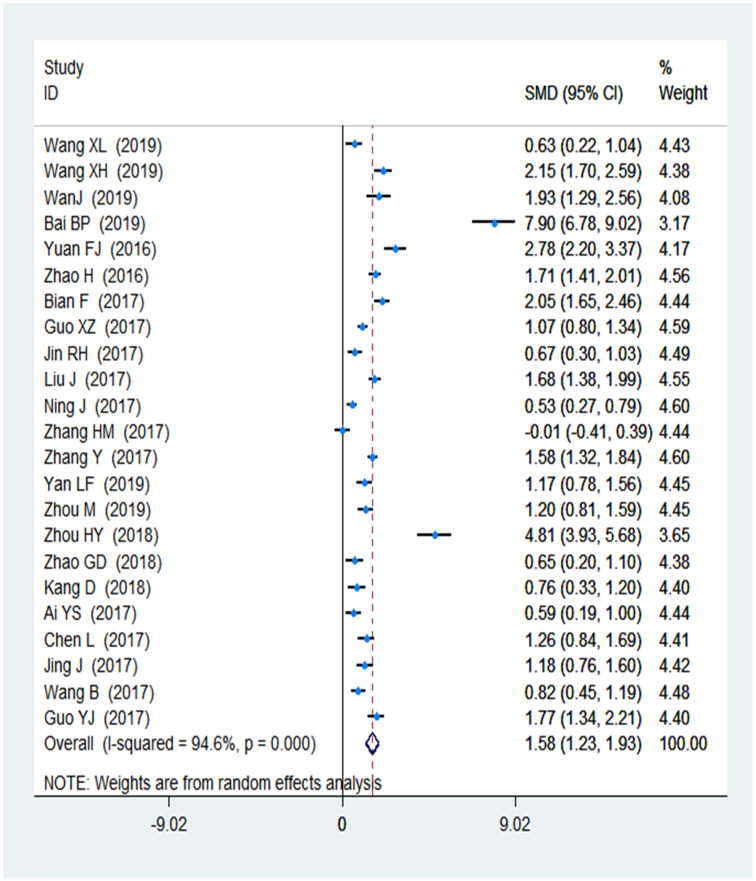
Forest plot of the nursing students' skills scores.

**Fig 6:**
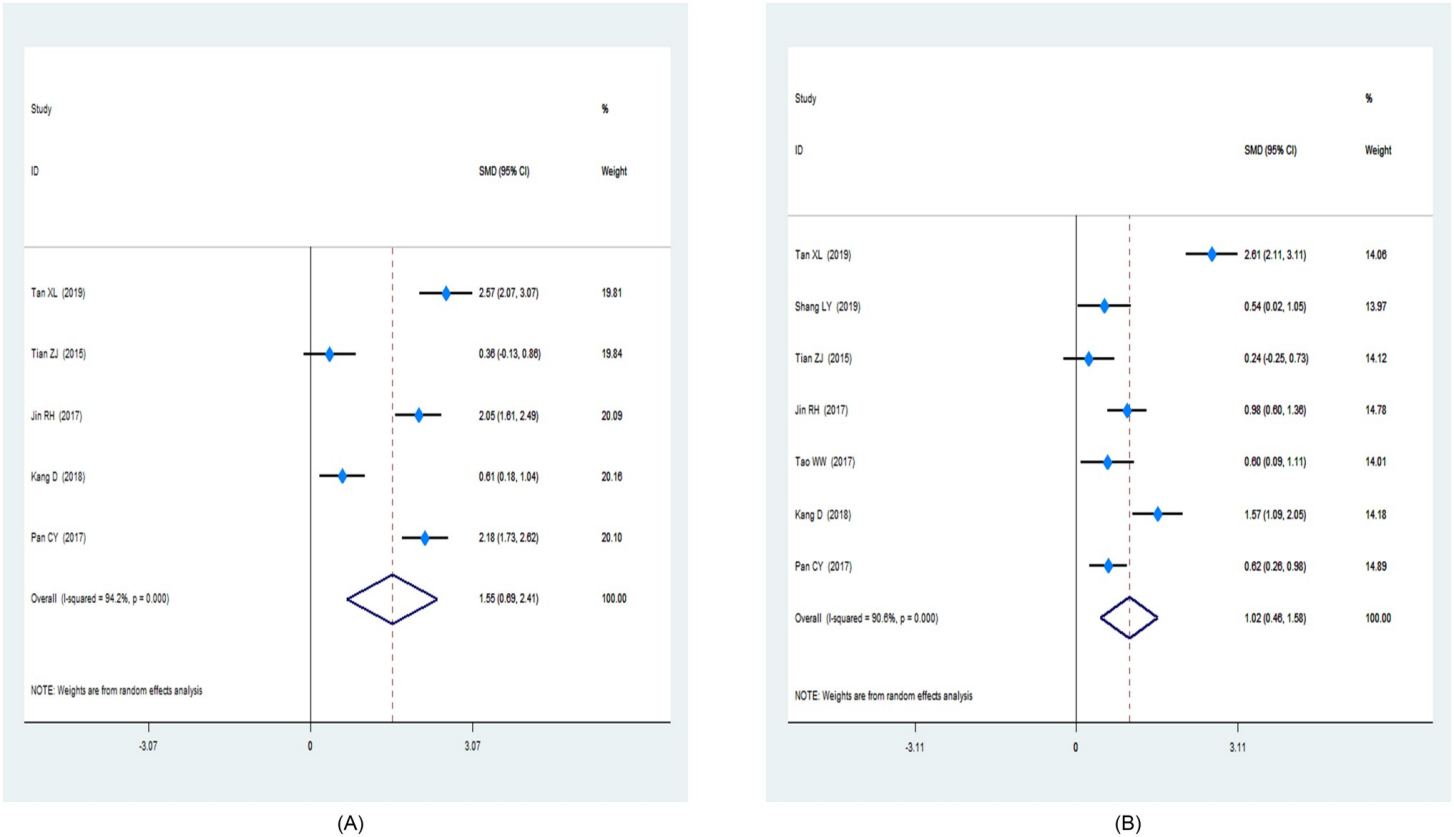
Forest plot of the nursing students’ feedback. (A): cooperative spirit; (B): self-management.

Based on the skill scores and student feedback, subgroup analyses were performed in this study. The skill scores were reported as a secondary outcome in 23 studies [26–28, 33–35, 37, 38, 41, 42, 45–54] (1,549 and 1,539 in the FC and LBL groups, respectively). The mean of the skill scores in the FC was 1.58 compared to that in the traditional classroom (95% CI: 1.23–1.93; *P* < 0.001). A forest plot of the nursing students’ skill scores is shown in Fig 5. The results of students’ feedback, as it pertains to cooperative spirit and self-management, were represented in two respective forest plots respectively (Fig 6). In general, student feedback from the FC group was better than that from the LBL group.

### How the FC affected different characteristics

Publication year, educational background, and subject were each considered a subgroup. The scores for different characteristics were analyzed as subgroups, and the results are shown in Table 2. Analysis revealed that in some segments of the subgroups, the theoretical scores of the FC group were higher than that of the LBL group. Moreover, there was significant heterogeneity among the different subgroups (*I*^*2*^ > 50%), indicating that these three characteristics were irrelevant to heterogeneity. This meta-analysis was limited by low reliability because these subgroups were unable to reduce the level of heterogeneity.

**Table 2:** Subgroup analyses of the nursing outcomes stratified by the various contexts.

Characteristics	n	Pooled Cohen's df (95% confidence interval)	*P*	Heterogeneity test		*P* (Egger's test)	*P* (Begg's test)
				*I*^*2*^	*P*		
Number					0.000		
<100	14	0.62 (0.49, 0.75)	0.000	95.10%		0.002	0.352
≥100	20	1.20 (1.12, 1.27)	0.000	95.30%		0.003	0.002
Publish year					0.000		
2019	9	1.75 (0.92, 2.58)	0.000	96.90%		0.017	0.012
2018	3	1.99 (-0.38, 4.35)	0.000	95.40%		0.027	0.117
2017	17	0.94 (0.60, 1.36)	0.000	93.00%		0.446	0.249
2016	4	2.16 (1.18, 3.14)	0.000	96.80%		0.174	0.111
2015	1	0.34 (-0.16, 0.83)	-	-	-	-	-
Educational background					0.000		
undergraduate	23	1.42(1.02, 1.82)	0.000	96.20%		0.109	0.162
higher vocational education	11	1.14 (0.67, 1.60)	0.000	92.50%		0.006	0,938
Duration of intervention					0.000		
availability	12	0.98 (0.90, 1.05)	0.000	96.40%		0.943	0.672
no availability	22	1.33 (1.19, 1.47)	0.000	94.70%		0.000	0.100
Subject							
basic nursing	9	0.86 (0.73, 0.99)	0.000	97.10%	0.000	0.532	0.117
surgical nursing	4	1.15 (0.95, 1.35)	0.000	94.70%		0.055	0.042
health assessment	2	1.59 (1.27, 1.91)	0.000	95.10%		0.317	-
obstetrics and gynecology nursing	3	0.88 (0.71, 1.05)	0.000	87.60%		0.117	0.356
internal medicine nursing	3	0.81 (0.57, 1.05)	0.444	-		0.117	0.382
other	13	1.21 (1.11, 1.32)	0.000	96.20%	0.000	0.239	0.180

### Sensitivity analysis

**Fig 7:**
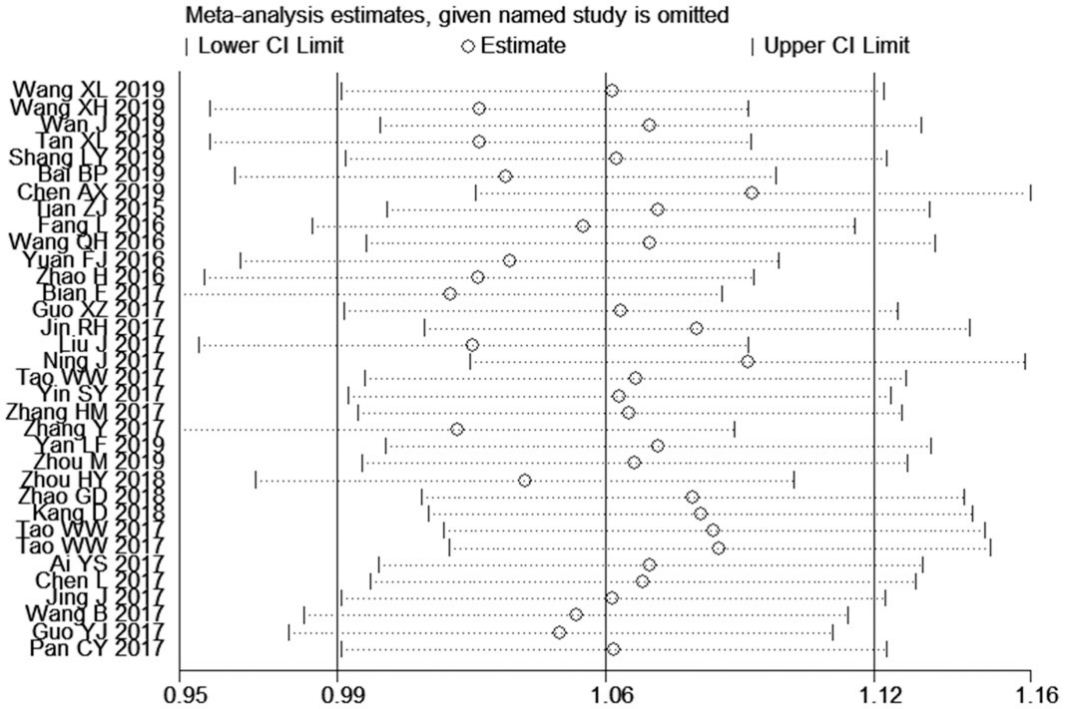
Sensitivity analysis of the nursing students' skills scores.

Sensitivity analyses were conducted in this meta-analysis. To assess the statistical stability of the results, the impact of a single dataset on the combined results was shown after removing a single study. The results of the sensitivity analyses of the FC and LBL are shown in Fig 7. For the flipped classroom, there were no excluded studies. The results pertaining to the theoretical scores were statistically stable (Estimate = 1.04, 95% CI: 0.98–1.11, *P* < 0.001).

## Discussion

This 34-study meta-analysis of summarized Chinese nursing students’ relevant outcomes, including theoretical test and skill scores. The results of this study indicated that the FC mode produced a significantly higher theoretical scores than the LBL mode, with a mean theoretical knowledge score of 1.33 for the former. Moreover, the mean of the flipped classroom skill scores was 1.58 times higher than that of the traditional classroom. These results are consistent with previous results in pharmaceutical [56], dental [18], and ophthalmology [57] education. Students who are new to the flipped classroom require a certain adaptation period during which they can familiarize with and prepare to embrace this teaching method [58]. Furthermore, the results of this review showed that effects of the FC on undergraduate and higher vocational students were almost the same and that both which were better than LBL teaching. It meant that using the FC was effective in different educational backgrounds. There are some explanations for the more active student learning and the better effect of the FC over the LBL. One explanation is that the FC allows students to master content at their own pace while browsing online materials. Since students can only focus on a lecture for about 10 minutes before their attention is reduced, using online materials can give them the flexibility to rest as needed [59]. Another explanation, classroom activities are designed to allow students to focus on applying the content to better understand the materials being taught [56]. These activities can be completed individually or in peer teams, thus shifting the teacher’s role from source of knowledge to promoter of student learning [56]. Moreover, in the FC, class time is full use for students to apply, analyze, and evaluate the knowledge [60]. As such, the FC has been proven to effectively improve students’ motivation, satisfaction, academic performance, and classroom engagement [61–63].

Some scholars have recognized the traditional classroom’s limitations; hence, it is very important to change or improve LBL teaching, given that faculty members teaching experiences and the teaching methods that they choose influence students’ learning feedback [58]. Inadequate teaching leads to low efficiency among teachers, which among teachers in students’ inattention during class. By this measure, many scholars have attempted to evaluate the new teaching mode ‘s effect on Chinese students. The FC is one of several feasible teaching modes. Based on various massive open online course (MOOC) platforms, the FC mode can be applied to numerous curricula, with students choosing an appropriate MOOC video for self-learning toward the overarching goal of improving their learning efficiency. Furthermore, pre work, such as reading and writing, is essential [64]. In this meta-analysis, the FC improved students’ theoretical performance, but its effect was not as beneficial with respect to skill performance Therefore, instructional designers should make full use of MOOCs to further improve students’ theoretical performance.

As one of the most populous countries in the world, with unmet needs of nurse number and various quality of nursing education, China needs to equip itself to deal with a large complex healthcare system [65]. Besides, in China, the study load and duration of nursing programs are high. Furthermore, as an undergraduate, every nursing student has to complete ten semesters over 5 years to finish complete various courses, including surgical, critical care, and pediatric nursing, and more. Moreover, Lee et al. [66] illustrated that nursing students need to study not only nursing but also other medical courses such as medical physics, microbiology, parasitology, and preventive medicine. In addition, nursing students need participate in clinical practice in their final academic year [66]. However, Limited content can be learned in the restricted time, and it’s very difficult for students to master a large volume of professional knowledge in such a short time. It is therefore crucial to explore an efficient teaching mode. This meta-analysis suggests that the flipped teaching mode significantly increases students’ theoretical scores, comparing favorably with the LBL mode. In future, the FC mode is worth using in nursing education.

Apart from the FC mode, there are other novel teaching modes, such as problem-based learning (PBL), case-based learning (CBL), and team-based learning (TBL). PBL mode concentrates on the process of students discovering and solving problems [67]. In CBL mode, students focus on the key point and solve real-world cases with facilitators’ guidance [68]. TBL focuses on solving problems through group cooperation and discussion [69]. A variety of teaching models have been used in various areas of professional teaching. For instance, Hu [70] applied the mixed FC and PBL teaching mode Similarly, Elangovan et al. [71] integrated PBL and CBL in dental education. Moreover, a mixed mode that combined the FC with team-, case-, lecture-, and evidence-based learning in ophthalmology teaching yielded only improved independent learning among students, but also an awareness of cooperation and competition [72].

There are some strengths in this meta-analysis. Theoretical score is considered as the first outcome and skill score as second outcome to compare the effects of the FC and LBL in this review. Besides, The FC mode in nursing education is popular and has been applied to ensure its effect. In this meta-analysis, all studies were RCTs and had different educational backgrounds, which were examined to judge whether different educational backgrounds would affect the effect of the new teaching mode.

This meta-analysis had several limitations. First, with regard to the geographical generalizability of the results, only Chinese articles met the screening criteria and were included in this study; thus, this study is only able to evaluate the FC’s implementation effect in China or Asia, as it would be difficult to a make such an assessment in other regions. Second, the included studies had significant heterogeneity, which may have reduced the reliability of the analysis. Furthermore, study quality was uneven because of different teaching resources, levels and evaluation standards in different schools. Third, the funnel plot for theoretical scores revealed asymmetry, indicating a publication bias.

## Conclusion

The results of this meta-analysis suggest that compared to the LBL teaching method, FC mode significantly improved Chinese nursing students’ theoretical scores. However, high heterogeneity and publication bias were present, which may have influenced the reliability of the FC effect in the context of nursing teaching. High-quality, multi-regional studies should be feature in future analysis.

## Supporting information

S1 Table Data of all included studies. (DOCX)

S1 Checklist PRISMA 2009 checklist. (PDF)
