# Supplementary material for: Flipped classroom improves nursing students’ theoretical learning in China: A meta-analysis
Source: PLoS One. 2020 Aug 27;15(8):e0237926. doi: 10.1371/journal.pone.0237926 (PMC7451570; doi:10.1371/journal.pone.0237926)
Supplement: S1 Table — (DOCX) [file pone.0237926.s001.docx]

**S1 Table. Data of all included studies**

| Study ID | Publication year | FC | | LBL | |
| --- | --- | --- | --- | --- | --- |
|  |  | TS (Mean±SD) | SS (Mean±SD) | TS (Mean±SD) | SS (Mean±SD) |
| Wan J [24] | 2019 | 75.17±8.86 | 92.45±1.09 | 71.54±11.27 | 90.35±1.09 |
| Shang LY [25] | 2019 | 81.77±4.60 |  | 76.93±6.13 |  |
| Wang XL [26] | 2019 | 77.57±8.88 | 86.00±4.59 | 68.07±10.28 | 82.90±5.27 |
| Bai BP [27] | 2019 | 80.22±1.84 | 90.70±1.53 | 75.31±2.11 | 76.36±2.05 |
| Wang XH [28] | 2019 | 61.43±1.21 | 14.50±0.41 | 54.12±1.31 | 13.53±0.51 |
| Tan XL [29] | 2019 | 81.43±2.36 |  | 73.10±2.34 |  |
| Yan LF [45] | 2019 | 83.24±4.56 | 90.50±4.38 | 79.65±6.24 | 84.69±5.42 |
| Zhou M [49] | 2019 | 92.80±7.03 | 91.70±6.21 | 88.00±4.31 | 85.60±3.65 |
| Chen AX [30] | 2019 | 84.56±8.76 |  | 79.60±9.61 |  |
| Zhang HY [50] | 2018 | 87.17±2.54 | 85.20±1.74 | 71.54±2.75 | 74.19±2.73 |
| Zhao GD [51] | 2018 | 37.61±3.56 | 37.40±3.65 | 37.21±2.45 | 35.02±3.54 |
| Kang D [52] | 2018 | 76.77±8.87 | 83.40±4.12 | 75.68±9.38 | 80.12±4.57 |
| Tao WW [31] | 2017 | 76.32±7.63 |  | 71.63±7.48 |  |
| Tao WW [31] | 2017 | 68.36±6.78 |  | 72.35±8.96 |  |
| Tao WW [31] | 2017 | 70.38±7.36 |  | 75.24±8.53 |  |
| Yin SY [32] | 2017 | 80.43±10.60 |  | 70.75±11.16 |  |
| Ai YS [46] | 2017 | 88.35±8.62 | 85.90±7.57 | 83.32±6.86 | 81.29±8.01 |
| Chen L [47] | 2017 | 82.78±4.05 | 86.60±4.27 | 79.36±5.31 | 80.63±5.12 |
| Jing J [48] | 2017 | 80.78±5.60 | 80.50±7.28 | 74.78±6.51 | 72.38±6.44 |
| Zhang HM [33] | 2017 | 68.30±9.42 | 81.60±3.32 | 62.68±1.28 | 81.64±4.65 |
| Wang B [53] | 2017 | 49.22±1.02 | 65.30±11.27 | 39.77±10.43 | 54.63±14.71 |
| Jin RH [34] | 2017 | 79.33±8.90 | 84.00±7.32 | 75.36±11.10 | 77.99±10.48 |
| Guo YJ [54] | 2017 | 86.98±6.52 | 84.40±7.49 | 77.24±6.48 | 72.00±6.50 |
| Bian F [35] | 2017 | 56.28±3.73 | 23.50±3.67 | 43.07±4.53 | 15.72±3.94 |
| Pan CY [55] | 2017 | 80.75±6.48 |  | 74.97±5.10 |  |
| Liu J [36] | 2017 | 88.06±2.43 | 88.70±2.59 | 83.68±2.54 | 84.37±2.49 |
| Guo XZ [37] | 2017 | 85.46±6.06 | 93.30±2.73 | 78.25±8.21 | 89.75±3.86 |
| Ning J [38] | 2017 | 85.75±10.50 | 89.40±14.86 | 79.36±12.67 | 81.36±15.63 |
| Zhang Y [39] | 2017 | 56.01±2.42 | 36.10±2.83 | 52.04±2.61 | 32.15±2.14 |
| Fang L [40] | 2016 | 81.35±6.39 |  | 73.20±6.37 |  |
| Yuan FJ [41] | 2016 | 38.78±1.15 | 56.50±2.25 | 32.45±1.30 | 50.25±2.22 |
| Zhao H [42] | 2016 | 87.86±2.33 | 88.80±2.61 | 83.71±2.58 | 84.36±2.52 |
| Wang QH [43] | 2016 | 86.21±3.67 |  | 82.35±4.29 |  |
| Tian ZJ [44] | 2015 | 72.66±5.24 |  | 70.97±4.78 |  |
